# Nb_2_CT_x_ MXene reinforcement stimulated microstructure and mechanical properties of magnesium

**DOI:** 10.1038/s41598-023-41067-8

**Published:** 2023-08-31

**Authors:** Ogunlakin Nasirudeen Olalekan, S. Fida Hassan, Amir Al-Ahmed, Nasurullah Mahar, Saheb Nouari

**Affiliations:** 1 Interdisciplinary Research Center for Advanced Materials, King Fahd University of Petroleum and Minerals, 31261 Dhahran, Saudi Arabia; 2 Department of Mechanical Engineering, King Fahd University of Petroleum and Minerals, 31261 Dhahran, Saudi Arabia; 3 Interdisciplinary Research Center for Renewable Energy and Power Systems, King Fahd University of Petroleum and Minerals, 31261 Dhahran, Saudi Arabia; 4 Department of Chemistry, King Fahd University of Petroleum and Minerals, 31261 Dhahran, Saudi Arabia

**Keywords:** Engineering, Materials science

## Abstract

In this study, Nb_2_CTx MXene reinforced commercially pure magnesium composite was processed using traditional blend-press-sinter technique. The added one volume percentage of Nb_2_CTx MXene was fairly dispersed around the magnesium particles despite having sporadic clustering. Nb_2_CTx MXene reinforcement was stable and developed defect free strong interfacial bonding with the magnesium matrix. The small amount of chemically compatible and thermally stable Nb_2_CTx MXene reinforcement was successful in enhancing the bulk hardness and compressive yield strength, compressive strength, ductility and fracture toughness of the commercially pure magnesium.

## Introduction

Magnesium (Mg) is one of the abundantly available elements in the earth’s crust and seawater. Magnesium based materials are well known for their low density and excellent mechanical properties, making them an attractive candidate for various engineering applications^1–4^ ranging from aerospace, defense, automobile, sports to consumer product. However, their inherent poor corrosion resistance and low stiffness, strength and ductility often limit their considerable use in structural application. In recent years, researchers have investigated the use of extremely fine reinforcing materials, including different oxide ceramics, carbon nanotube and graphene, to enhance the mechanical properties of Mg alloys^5–10^. However, one of the most promising reinforcement materials among them is MXene^11–13^.

MXenes are a new family of two-dimensional (2D) materials composed of transition metal carbides and nitrides with a formula of M_n+1_X_n_T_x_, where M is a transition metal, X is carbon or nitrogen, T is a surface termination group (such as O, OH, F and/or Cl), and n is an integer^14–16^. The n layers of carbon or nitrogen atoms interleaved into n + 1 layers of transition metals in the MXene and are produced by selectively etching the A (mainly elements from IIIA or IVA group of periodic table) element of MAX phases using hydrofluoric acid or other strong acids. The resulting MXene materials has a layered structure and that can be easily delaminated into thin two-dimensional sheets. The A elements act as glue to keep transition metal carbides and/or nitrides together in the layered MAX phase structure. Researchers has developed more than sixty MXene composition^15, 17^ and computationally predicted more than hundred potential MXene composition^15, 18^ since the invention of first MXene, i.e., Ti_3_C_2_T_x_, in 2011^19^. Strong chemical stability, efficient electromagnetic wave absorption capacity, high electrical conductivity and excellent mechanical properties made the MXene promising reinforcement materials for various applications, including the metal matrix composites.

In recent years, researchers have investigated the use of MXene as a reinforcing materials in various metal including the aluminum^20, 21^, copper^22–25^, nickel^26^, titanium^27^ and magnesium alloy^11, 12^ and produced metal matrix composites with a wide range improved metal properties. The addition of MXene to magnesium alloy ZK61 using a powder metallurgy method has significantly improved the mechanical properties including compressive yield strength, ultimate compressive strength and ductility. However, understanding the effect of wide range of the available MXene on the large number of known magnesium based materials cannot be predicted from the reported one formulation of MXene reinforced magnesium alloy, i.e., ZK61-Ti_3_C_2_T_x_. Hence, the initiative has been taken in study to reinforce the commercially pure magnesium with in-house developed Nb_2_CTx MXenes using traditional blend-press-sinter (BPS) powder metallurgy process. The physically blended magnesium-Nb_2_CTx MXenes composite powder was compacted and sintered to study the effect of MXene reinforcement on the microstructure and the ensued mechanical properties of commercially pure magnesium.

## Experimental procedure

### Raw materials

**Figure 1:**
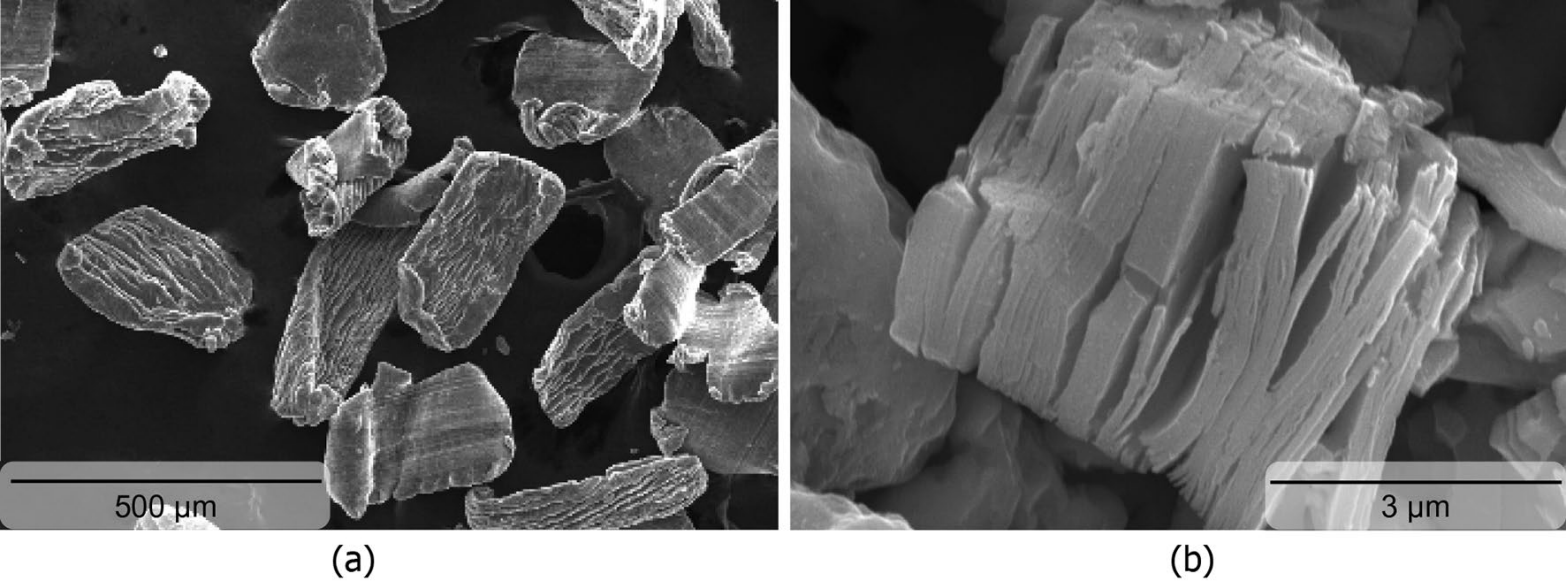
Scanning electron micrographs showing particles of (**a**) commercially pure magnesium, and (**b**) Nb_2_CT_X_ MXene, respectively.

In this study, magnesium particles with 98.5+ % purity and an average size range of 60–300 μm (from Merck KGaA, Germany) reinforced with Nb_2_CTx MXenes. Morphology of the raw materials are shown in Fig. 1. The Nb_2_CTx MXenes were synthesized from their Nb_2_AlC MAX Phase precursor using a previously reported method with minor modification^28^. In the MXene synthesis process, 1 g of Nb_2_AlC MAX phase gradually added to a mixed acid etchant solution (i.e., 5 ml of 48% HF and 15 ml of 12 M HCl) in a Teflon bottle at 35 °C. This mixture was stirred at 500 rpm for 10 min in an ice bath to avoid premature metal oxidation and subsequently for 72 h in an oil bath at 50 °C. The resulting black-colored suspension was centrifuged at 6000 rpm to collect the sediment product was collected while the supernatant liquid (i.e., solution of dissolved slats and metal) was decanted. The prepared multilayered Nb_2_CTx MXene was treated with triethylamine (TEA) intercalant for 6 h under constant stirring. The product was centrifuged at 2500 rpm followed by washing by water–ethanol mixture (1:1) in sonication bath. The resulting Nb_2_CTx MXene suspension was vacuum dried at 110 °C for 2 h for their application as reinforcement.

### Powder processing of composite

To create a composite material, magnesium powder was mixed with Nb_2_CTx MXene particles using a 8000D Dual Mixer/Mill machine. The machine was operated at a speed of 200 rpm for 30 min without any hard balls. The resulting blended powders were then cold compacted into cylindrical billets with a diameter of 10 mm using a uniaxial hydraulic press under a pressure of 450 MPa for 2 min. The cold compacted billets were sintered at 550 °C (i.e., ~ 0.9 T_mp_, of pure magnesium) for 90 min in an inert argon gas environment using an electrical resistance heating tube furnace (MTI GSL-1700X, MTI Corporation, USA). The sintering process was followed by furnace cooling of the sintered samples. Inert argon gas was used as the sintering environment to avoid the oxidation of the magnesium powder.

### Characterization of sintered composite

Density (ρ) of the sintered Mg-Nb_2_CTx MXene composite samples was measured in accordance with Archimedes' principle^29^. The samples were weighed in Mettler Toledo model AG285 Electronic balance with an accuracy of ± 0.0001 g and distilled water was used as the immersion fluid.

Microstructural characterization was conducted on the metallographically prepared Mg-Nb_2_CTx MXene composite samples to investigate Nb_2_CTx MXene distribution pattern and their interfacial integrity with commercially pure. QUANTA 250 FEG- FEI field emission scanning electron microscope (FESEM) equipped with energy dispersive spectroscopy (EDS) was used in this purpose.

Mechanical characterization of the Mg- Nb_2_CTx MXene composite was conducted to investigate the effect of Nb_2_CTx MXene on the macrohardness and compressive behavior of commercially pure magnesium. Macrohardness was measured in Rockwell 15 T superficial scale using a Rockwell hardness tester in accordance with ASTM E18-03 standard. Compressive behavior evaluated on cylindrical samples in accordance to the ASTM E9-09 (2018). Instron 3367 machine used with a crosshead speed of 0.050 mm/min during the compressive tests.

## Results and discussion

The traditional powder metallurgy route, commonly known as the blend coat-press-sinter (BPS) method, was used to process an Nb_2_CTx MXene reinforced commercially pure magnesium composite. Synthesis process involved physically dry coating the commercially pure magnesium powder particles with 1 vol% of Nb_2_CTx MXene particles. The processed billets of the composite and unreinforced magnesium, both in compact and sintered forms, displayed processing-induced defects free smooth surface and were of the intended size and shape. The effectiveness of the processing parameters was justified by the absence of any dimensional distortion and/or surface cracks and/or orange peel and/or oxidation marks in both the as compact and as-sintered forms of the billets. Effectiveness of the high compaction pressure (i.e., 450 MPa) and high sintering temperature (i.e., 0.9T_m_, k) used in the processing is also justified by the almost near-dense density values (see Table 1) of the Nb2CTx MXene reinforced magnesium (99.6%) and the unreinforced reference material (99.1%). It has to be noted that the compaction pressure used in this study was much higher than the tensile strength of commercially pure magnesium and hence was capable in inducing significant plastic flow of the particles to enhance the inter-particle surface contact by effectively removing the inter-particle micropores. The extended enhanced inter-particle surface contact played significant role in producing the near-dense Nb_2_CTx MXene reinforced and the unreinforced magnesium during the subsequent high temperature sintering process^30, 31^.

**Table 1:** Results of density and porosity of Nb_2_CT_X_ MXene reinforced magnesium composite.

Material	Density (g/cm^3^)			Porosity^d^ (%)
	Theo.^a^	Expt.^b^	Relative^c^	
Mg-Nb_2_CT_X_	1.797	1.790	99.59	0.41
Mg	1.738	1.722	99.10	0.90

^a^ρ using rule-of-mixture.

^b^ρ using Archimedes’ principle.

^c^ρ = experimental density/theoretical density.

^d^Porosity = [(ρ_Theoretical_ – ρ_bulk_)/(ρ_Theoretical_ – ρ_air_)] × 100.

**Figure 2:**
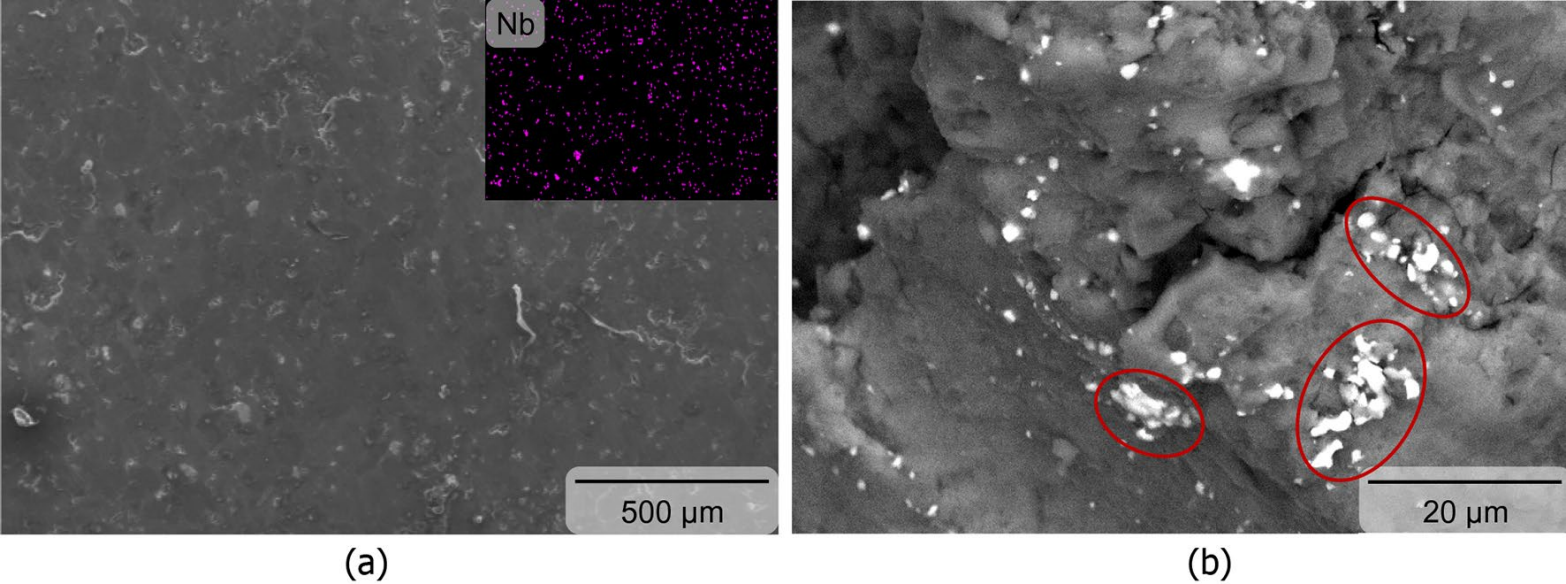
Scanning electron microscopy showing (**a**) dispersion of Nb_2_CT_X_ MXene particles (inset: Nb mapping) as (**b**) necklace form (white particles) around magnesium particles with presence sporadic clusters (red circles) in fracture surface), respectively.

Microstructural study on the Nb_2_CTx MXene reinforced commercially pure magnesium revealed that the reinforcement particles were dispersed in the commercially pure magnesium (see Fig. 2a) mostly in the necklace form around the magnesium particles with presence of some sporadic high-particles concentration area in the matrix (see Fig. 2b).

**Figure 3:**
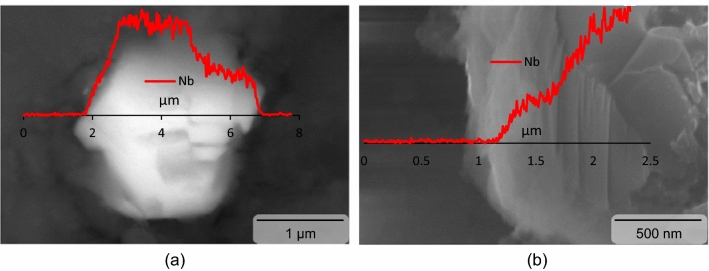
Scanning electron microscopy associated with energy dispersive X-ray spectrometry showing Mg-Nb_2_CT_X_ MXene particles interfacial morphology in as sintered (**a**) and fractured (**b**) form, respectively.

The relatively thin distribution pattern of Nb2CTx MXene particles in the sintered magnesium can be attributed to the implementation of suitable blending parameters. However, some degree of segregation of Nb_2_CTx MXene particles in the physically blended magnesium-Nb_2_CTx MXene powder was also anticipated due to the large difference in the density values between Nb_2_CTx MXene particles (i.e., 7.65 g/cm^3^)^32^ and magnesium particles (i.e., 1.74 g/cm^3^)^33^. Moreover, cluster of raw MXene particles may typically breaks in polar organic solution^34^ which may have adverse effect on the elemental magnesium particles^35^ and hence could not use in the Nb_2_CTx MXene-magnesium blending stage. Microstructural study also revealed the presence of defect-free interface between Nb_2_CTx MXene reinforcement particles and magnesium matrix (see Fig. 3a). The Nb_2_CTx MXene-magnesium matrix interfacial integrity was assessed in terms of micro-voids and reaction product. Magnesium has apparently good compatibility with the high temperature Nb_2_CTx MXene. There was no identifiable Nb_2_CTx MXene-magnesium reaction products present in the interface and could be attributed to the absence of any mutual solubility and/or stable reaction product between magnesium and niobium or carbon^36^. The good compatibility and strong interfacial integrity of the Nb_2_CTx MXene reinforcement particles with the magnesium was also supported by the reinforcement particle fracture (see Fig. 3b), instead of pull out or debonding, under applied compressive stress. However, there was no apparent effect of Nb_2_CTx MXene particles on the grain morphology of the magnesium matrix and it could apparently due to the clustering of the reinforcement.

Presence of negligibly minimal porosity was observed in the Nb_2_CTx MXene particles reinforced magnesium matrix (see Table 1) and that could be attributed to the cumulative effect of the very high compaction pressure, longer high temperature sintering and apparent good interfacial compatibility between magnesium and Nb_2_CTx MXene particles.

Mechanical behavior of sintered Nb_2_CTx MXene particles reinforced commercially pure magnesium was studied in terms of macrohardness and compression-to-failure compression test (see Table 2). Hardness of the of the commercially pure magnesium matrix was significantly increased (i.e., 88%) and can be attributed to the presence of relatively harder Nb2CTx MXene particles as has been noticed earlier^11, 12^.

**Table 2:** Results of hardness and compressive properties of Nb_2_CT_X_ MXene reinforced magnesium composite.

Material	Hardness (HR15T)	0.2%YCS (MPa)	UCS (MPa)	Elongation (%)	Energy absorb (MJ/m^3^)
Mg-Nb_2_CT_X_	15 ± 2.8 (88↑)	92 ± 3 (11↑)	130 ± 3 (16↑)	11.17 ± 0.95 (9↑)	11.29 ± 1.1 (27)
Mg	8 ± 2.6	83 ± 3	112 ± 2	10.22 ± 0.05	8.90 ± 0.80

() represents increase in %values due to Nb_2_CT_X_ reinforcement.

**Figure 4:**
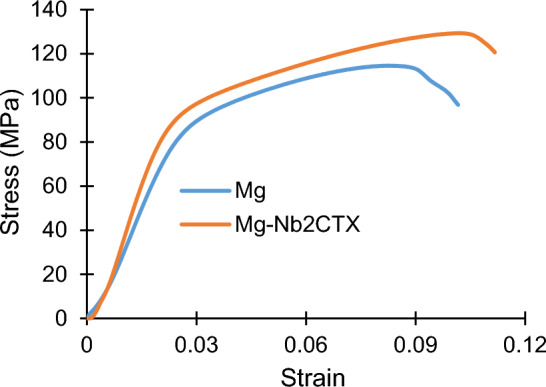
Graphs showing compressive stress–strain behavior of sintered Nb_2_CT_X_ MXene particles reinforced magnesium.

Nb_2_CTx MXene particles simultaneously enhanced (see Fig. 4 and Table 2) the compressive strength (11% and 16% for yield and ultimate, respectively) and ductility (9%) as well the toughness (27%) of commercially pure magnesium. Yield strength of metallic materials represent the level of difficulty to initiate the permanent deformation process, while the dislocation and/or tensile twin motion activates the plastic deformation process in magnesium-based materials. Under externally applied compressive stress, the sintered magnesium can yield very easily since it requires relatively lower Schmid Factor^37, 38^. It has to be noted that sintered magnesium structure develops random textures similar to the case of cast microstructure and the textured microstructure assist in the easy activation of deformation tensile twin leading to relatively lower compressive yield strength (i.e. 92 MPa, which is 11% higher than that of unreinforced magnesium). The advancing dislocation slip and deformation tensile twin, the latter is dominating deformation mode under compressive stress, pile-up at the gain boundary prior to transfer to next grains. Beside the grain boundary, the Nb_2_CTx MXene particles reinforcement present at the magnesium inter-particle space also restricts the advancement of the dislocation slip and deformation tensile twin and contribute to the strain hardening of the reinforced magnesium after yield. Strain hardening led to a reasonable compressive strength in the reinforced magnesium (130 MPa, which is 38 MPa higher than the yield strength but 16% higher than the compressive strength of unalloyed magnesium). Thermally stable non-reactive hard nonmetallic particles as reinforcement typically improves the strength of magnesium through various strengthening mechanisms including matrix grain refinement, mismatch in elastic modulus and coefficient of thermal expansion between matrix-reinforcement, and Orowan strengthening^6, 7, 11, 12, 29^. Among these strengthening mechanisms, yield strength of magnesium is most profoundly affected by the matrix grain refinement and that was not effectively exploited in this Nb_2_CTx MXene particles reinforced commercially pure magnesium. Clustering of the Nb_2_CTx MXene particles also limited the anticipated Orowan strengthening effect in the sintered magnesium matrix.

**Figure 5:**
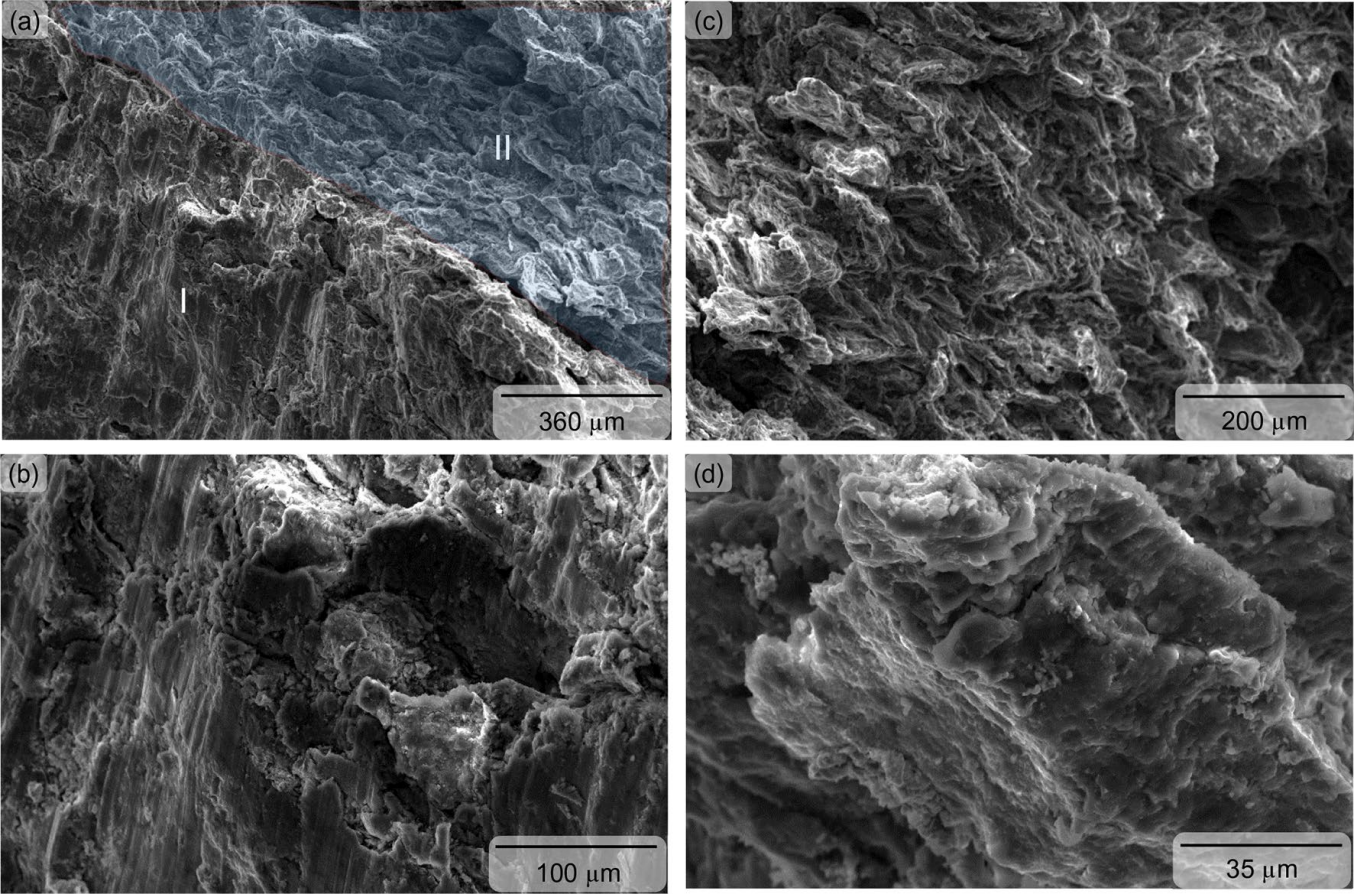
Fractographs showing the shear band as parallel line to fracture surface [zone I in (**a**), magnified in (**b**)] and wavy saw-toothed structure [zone II in (**a**), magnified in (**c,d**)] in sintered Nb_2_CT_X_ MXene particles reinforced magnesium.

**Figure 6:**
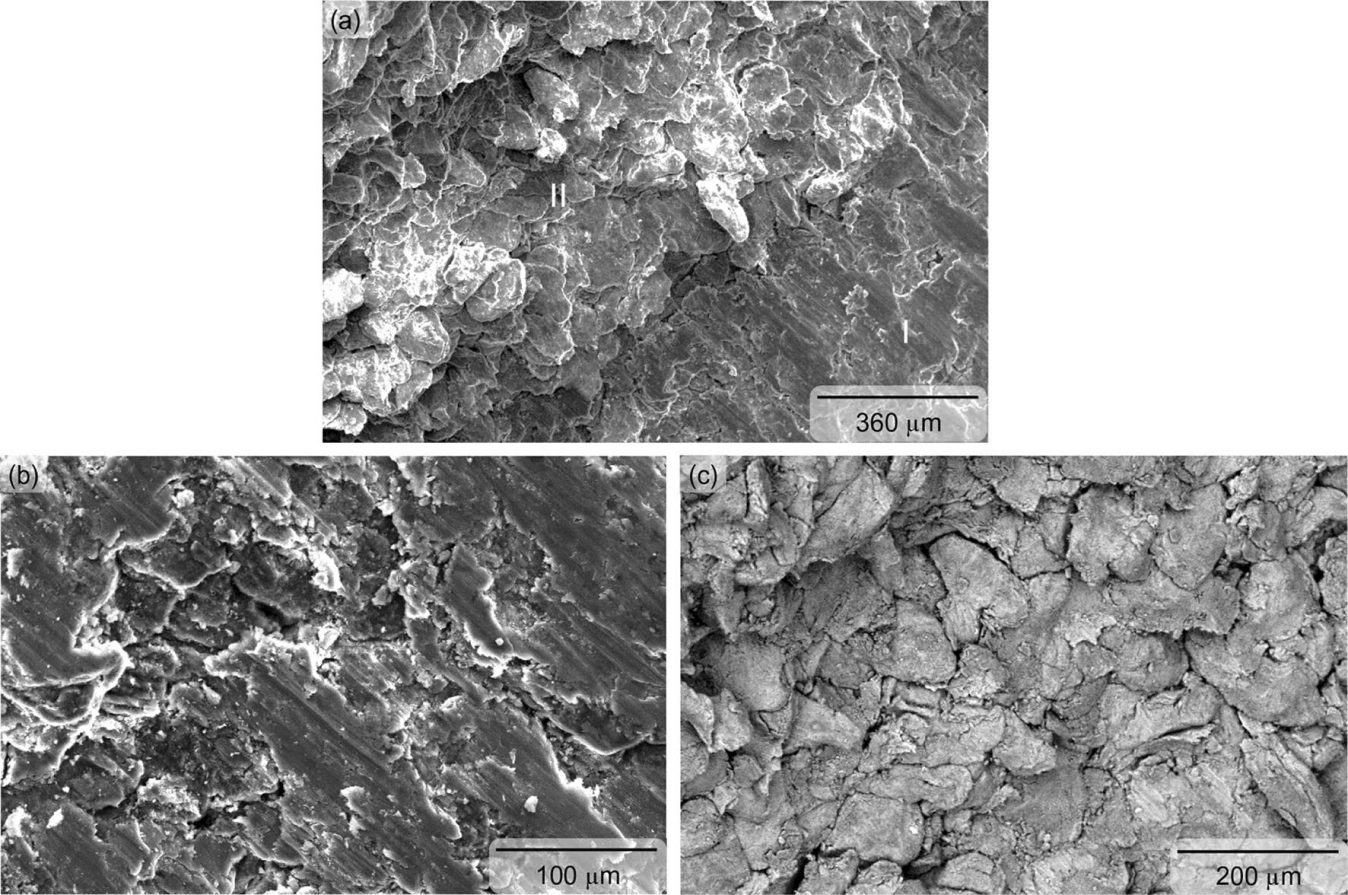
Fractographs showing the shear band as parallel line to fracture surface [zone I in (**a**), magnified in (**b**)] and intergranular fracture [zone II in (**a**), magnified in (**c**)] in sintered unreinforced magnesium.

The limit of compressive plastic deformation (i.e., ductility) of the sintered magnesium is determined by the total travel distance of the advancing dislocation slip and deformation tensile twin^39^, and was found to be superior (9% higher) when reinforced with the Nb_2_CTx MXene particles. Dominance of deformation tensile twin in the strain hardening and consequently in the compressive ductility is supported by the presence of shear-band parallel to the fracture surface of the Nb_2_CT_x_ MXene particles reinforced magnesium (see Fig. 5a, b). It was noticed that the shear-band was finer in the Nb_2_CT_x_ MXene particles reinforced magnesium when compared to the unreinforced magnesium (see Fig. 6a, b). The dislocation pile-up at the grain boundary apparently induced an additional mode of fracture, namely intergranular crack propagation in the unreinforced magnesium (see Fig. 6a–c). The dislocation pile-up was seemingly not dominating in the Nb_2_CT_x_ MXene particles reinforced magnesium fracture surface and the secondary fracture mode took the shape of saw-toothed shaped shear-band^40^ with the help of deformation tensile twin on the of (see Fig. 5a, c, d). The compression-to-fracture test also revealed that the capacity of energy absorption until fracture (measured by area under the stress–strain graph) of commercially pure magnesium was significantly enhanced (27%) due to the incorporation of Nb_2_CTx MXene particles as reinforcement (see Fig. 4 and Table 2).

## Conclusion

Traditional blend-press-sinter technique was capable in processing the commercially pure magnesium composite with one volume percentage of Nb_2_CTx MXene reinforcement. Nb_2_CTx MXene was chemically stable in magnesium matrix and has excellent interfacial integrity. Despite the reasonable dispersion of the Nb_2_CTx MXene in the magnesium matrix, the blending media and/or parameters used in this study was not fully efficient to disintegrate the reinforcement MXene clusters. The added small amount of Nb_2_CTx MXene reinforcement was competent in enhancing the bulk hardness and compressive characteristics (i.e., yield strength, compressive strength, ductility and fracture toughness) of the commercially pure magnesium matrix.

## Data Availability

Experimental data for this study can be obtained from corresponding author on reasonable request.
